# PP86 Development Of A Tool To Assist In The Identification Of Study Designs For The Purposes Of Health Technology Assessment

**DOI:** 10.1017/S026646232400254X

**Published:** 2025-01-07

**Authors:** Lavinia Ferrante di Ruffano, Katie Reddish, Emma Bishop, Deborah Watkins, Mary Edwards, Rachael McCool

## Abstract

**Introduction:**

As the most internally rigorous design, randomized controlled trials (RCTs) are the gold standard for assessing the efficacy and safety profile of interventions. Increasingly, health technology assessment (HTA) considers evidence from non-randomized studies. Guidance recommends synthesizing different study designs separately due to their different inherent biases/limitations. But when authors or reviewers misclassify studies, this could affect which studies are included and therefore have an impact on review results.

**Methods:**

We are conducting a methods project to (i) identify a clear study design classification system, (ii) explore whether its use produces consistent study design categorizations among reviewers, and (iii) iteratively improve the classification system. We performed a pragmatic web-based search for study design categorization tools and used the resulting schemas to develop a clear algorithm for use by reviewers of all levels of experience, specifically in reviews of treatment interventions. Next, we tested tool consistency and user experience by web-based survey in a small internal sample of reviewers, each independently using the system to categorize 18 published studies.

**Results:**

A median of seven reviewers (range four to eight) categorized each study. Rater agreement using the chart varied widely, with 100 percent agreement on the designs of three studies (17%), and at least 75 percent of reviewers agreeing on one design for nine studies (50%). The most common agreement was reached on RCTs and non-randomized controlled trials. The most common sources of disagreement were between different types of cohort studies and between case series and controlled cohort studies, largely due to inconsistent reporting. We also identified several improvements: the wording of prompt questions, the ordering of designs, and the addition of new elements.

**Conclusions:**

The classification system as initially designed led to too much variation in study design categorization to be useful. Consequently, we present a revised version that we now aim to evaluate in a larger sample of reviewers. Further research will also investigate whether using the tool would change the results of systematic reviews, using a small sample of published reviews.

